# Expression profiles of the pluripotency marker gene *POU5F1 *and validation of reference genes in rabbit oocytes and preimplantation stage embryos

**DOI:** 10.1186/1471-2199-9-67

**Published:** 2008-07-28

**Authors:** Solomon Mamo, Arpad Baji Gal, Zsuzsanna Polgar, Andras Dinnyes

**Affiliations:** 1 Genetic Reprogramming Group, Agricultural Biotechnology Centre, Szent Gyorgyi Albert u. 4, H-2100 Gödöllő, Hungary; 2 Faculty of Natural Sciences, Constantine the Philosopher University, Slovakia; 3 Molecular Animal Biotechnology Laboratory, Szent Istvan University, Pater K. u. 1, H-2103 Gödöllő, Hungary; 4 University College of Dublin, Lyons Research Farm, Newcastle Co. Dublin, Ireland

## Abstract

**Background:**

The surge in the number of gene expression studies and tendencies to increase the quality of analysis have necessitated the identification of stable reference genes. Although rabbits are classical experimental model animals, stable reference genes have not been identified for normalization. The aims of this study were to compare the expression profiles of the widely used reference genes in rabbit oocytes and preimplantation stage embryos, and to select and validate stable ones to use as reference.

**Results:**

Quantitative real time PCR method was used to evaluate 13 commonly used references (*Actb, Gapdh, Hprt1, H2afz, Ubc, Ppia, Eef1e1, Polr2a, Tbp, G6pdx, B2m, Pgk1*, and *Ywhaz*) and *POU5F1 (Oct4) *genes. Expressions of these genes were examined in multiple individual embryos of seven different preimplantation developmental stages and embryo types (*in vivo *and *in vitro*). Initial analysis identified three genes (*Ubc, Tbp*, and *B2m) *close to the detection limit with irregular expression between the different stages. As variability impedes the selection of stable genes, these were excluded from further analysis. The expression levels of the remaining ten genes, varied according to developmental stage and embryo types. These genes were ranked using the geNorm software and finally the three most stable references (*H2afz, Hprt1*, and *Ywhaz*) were selected. Normalization factor was calculated (from the geometric averages of the three selected genes) and used to normalize the expressions of *POU5F1 *gene. The results showed the expected expression patterns of the POU5F1 during development.

**Conclusion:**

Compared to the earlier studies with similar objectives, the comparison of large number of genes, the use of multiple individual embryos as compared to pools, and simultaneous analyses of *in vitro *and *in vivo *derived embryo samples were unique approaches in our study. Based on quantification, pattern and geNorm analyses, we found the three genes (*H2afz, Hprt1*, and *Ywhaz*) to be the most stable across developmental stages and embryo types, and the geometric averages of these genes can be used for appropriate normalization.

## Background

Preimplantation embryo development is a dynamic developmental process recognized by changes in the transcript type and quantity [1-3], cell number, total and poly (A) RNA contents [4,5]. These entire phenomena are known to be the basis for the changes in the shape, physiology and functions of the embryos leading to compaction, differentiation, implantation and further development. During this preimplantation period, however, about 15–50% of zygotes die, largely as a result of unknown factors [6]. Therefore, it is imperative to understand the dynamics and search for factors contributing to the losses. Moreover, elucidation of preimplantation development is critical for the management of infertility and refinement of assisted reproductive technology [7]. Gene expression profiles would yield insight into the complex molecular pathways controlling early development. However, the use of classical techniques [8-10] for embryo gene expression analyses was constrained by technical limitations and a dearth of starting materials. To overcome the problem, the production of embryos in large quantities is restricted by associated ethical and cost factors. We (and others) have described in detail the problems associated with the use of common RNA detection and analysis techniques for the application of preimplantation stage embryo studies [11,12].

Real time PCR is a sensitive quantitative method of choice that overcomes the potential sensitivity problems in the earlier classical analysis techniques [13], and detects more subtle changes in gene expression [14]. Its sensitivity allows working with a minimal amount of starting material, while still achieving an accurate quantification of poorly transcribed mRNAs [15]. It is a fast and reliable technique, provided care is taken in all the procedures [16]. The precautions associated with the use of this technique were described elsewhere [12] and, unless properly addressed, could lead to severe misinterpretation of the results [17,18]. To address the issue and account for variations, different normalization strategies were used, and the details were well reviewed [19]. Some researchers use exogenously added references in the form of synthetic RNA or globin RNAs [20,21]. However, this approach has been challenged for competing with endogenous sequences for enzyme and nucleotides during PCR, its demand for extra procedures and associated cost [9,19]. As a result, it has not been adopted widely. The use of endogenous reference genes (commonly known as housekeeping genes) to normalize the expression of gene(s) of interest is the most commonly used approach. Housekeeping genes are constitutively expressed to maintain cellular function, and they are presumed to produce the minimally essential transcripts necessary for normal physiology [22,23]. Despite their ease of use and the wider adoption of this approach, inconsistency in the type and number of reference genes used has made cross-study comparisons difficult (even for closely related studies) and interpretation of the results questionable. As a result, this area has been a hot topic and the focus of many journals publication in recent times.

Rabbits are classical experimental models with close similarity in developmental biology to large animals and humans [24]. They are also preferentially used in pulmonary, cardiovascular, metabolic disorder studies, and for antibody production and drug screening [25]. As an experimental animal for various models, different gene expression studies were carried out using rabbit embryos. Most of these studies [26,27] used a single normalizer gene that differed between studies. The uses of some of these genes (*Actb*, *Gapdh*, and ribosomal RNAs) were evaluated in different studies and criticized, due to the observed variations between treatment groups [28-31]. We have earlier published a similar mouse study [12]. However, stable genes have not been identified for use in rabbit preimplantation stage embryo gene expression studies. The aims of the current study were to compare the expression profiles of 13 widely used reference genes in rabbit oocytes and preimplantation stage embryos that were derived *in vivo *and produced *in vitro*, to select the most stable ones as reference and finally to validate them by using to normalize the expression of *POU5F1 *gene. The gene *POU5F1 *belongs to the POU domain family transcription factors that regulate the transcription of their target genes [32,33]. The rabbit *POU5F1 *gene was recently cloned [34], but its expression during rabbit embryo preimplantation stage has not been well characterized. Thus, we analyzed the expression of this gene in the oocyte and *in vitro *produced preimplantation stage rabbit embryos. To our knowledge, this is the first study in rabbit to compare the wide selection of reference genes both in the *in vitro *and *in vivo *derived embryo samples with a final validation of the selected genes.

## Results

### Sequence analyses and product confirmation

For the 14 genes used in this study, the sequences were referred from the databases (Table 1), and primers were designed from these sequences. After optimizing PCR conditions and amplification, the products were sequenced for confirmation. Based on the sequence analysis, all primers amplified the expected amplicon sizes (Table 2).

**Table 1:** Reference genes selected for the study

**Symbol**	**Gene name**	**Source sequences**	**Some of the References**
*Actb*	Actin, beta, cytoplasmic	X60733	2, 16, 17, 35
*Gapdh*	Glyceraldehyde-3-phosphate dehydrogenase	NM_001082253	2, 16, 17, 35
*Hprt1*	Hypoxanthine guanine phosphoribosyl transferase 1	ENSOCUG00000003186	2, 16, 17, 20
*H2afz*	H2A histone family, member Z	ENSOCUG00000001888	8, 12
*Ubc*	Ubiquitin	ENSOCUG00000001288	20, 34
*Ppia*	Peptidylprolyl isomerase A	ENSOCUG00000016662	10, 16, 17
*Eef1e1*	Eukaryotic translation elongation factor 1 epsilon 1	ENSOCUG00000012587	57, 12
*Polr2a*	Polymerase (RNA) II (DNA directed) polypeptide A	ENSOCUG00000017929	16
*Tbp*	TATA box binding protein	ENSOCUG00000007979	2, 16, 17, 20
*G6pdx*	Glucose-6-phosphate dehydrogenase X-linked	ENSOCUG00000007866	16, 21
*B2m*	Beta-2-microglobulin	ENSOCUG00000017117	10, 16, 17, 20
*Pgk1*	Phosphoglycerate kinase 1	ENSOCUG00000014726	17, 20
*Ywhaz*	Tyrosin 3-monooxygenase/tryptophan 5-monooxygenase activation protein, zeta polypeptide	ENSOCUG00000000734	2, 34
*POU5F1*	POU domain, class 5, transcription factor 1	NM_001099957	32

**Table 2:** Primer sequences and optimum PCR conditions for amplifying the products

Gene	Forward (5' to 3') Reverse (5' to 3')	Product size (bp)	Primer Used (nM)	Annealing Temp (T°)
*Actb*	TCCGCCGCCGGCCCACACT AGTCCTTCTGGCCCATGC	188	0.40 0.40	60
*Gapdh*	CAAGTTCCACGGCACGGTCA CTCGGCACCAGCATCACCC	118	0.40 0.40	68
*Hprt1*	ACGTCGAGGACTTGGAAAGGGTGTT GGCCTCCCATCTCCTTCATCACATC	96	0.40 0.40	68
*H2afz*	AGAGCCGGCTGCCAGTTCC CAGTCGCGCCCACACGTCC	85	0.20 0.33	59
*Ubc*	GTGACACCATCGAGAAT GACACCTCCCCTCAGAC	173	0.40 0.30	60
*Ppia*	TCCAGGGTTTATGTGCCAGGGTG CGTTTGCCATGGACAAGATGCC	137	0.30 0.30	68
*Eef1e1*	ACCGCAGAAGAGAAAGCCATAG AGCGATGTAGCCCATAGTAGAGGA	190	0.50 0.50	68
*G6pdx*	AGCCCGCCTCCACTGACTCC ACCACGTTGTCCGCCTGCAC	88	0.40 0.30	60
*Tbp*	GCTGAATATAATCCCAAGCGGTTTGC AAATCAGCGCTGTGGTTCGTGGCTCTC	73	0.30 0.30	68
*Polr2a*	AGACTTCTCGGCCCGCACTG ACTTGGCGCCTGGGTACTGG	176	0.40 0.40	68
*B2m*	GCTCCGTCTTGGGCTTG CGGATGAAACCCAGATACATAG	152	0.33 0.33	60
*Pgk1*	TGTTGGTCGGGCGAAGCAG CAGTGTCTCCACCGCCGATG	149	0.50 0.20	60
*Ywhaz*	GGTCTGGCCCTTAACTTCTCTGTGTTCTA GCGTGCTGTCTTTGTATGATTCTTCACTT	142	0.50 0.50	68
*POU5F1*	CGAGTGAGAGGCAACTTGG CGGTTACAGAACCACACACG	125	0.45 0.20	57

To amplify beta actin (*Actb) *gene, we designed primers from 3 different sequences (AY598932, AF404278 and X60733), optimized and compared them. However, the current primer (design from X60733 sequence) gave the best amplification results. It has also the best beta actin similarity hits with orthologs. Although the source sequence was identified as gamma non-muscle actin in the NCBI, and as actin, alpha skeletal in the Ensembl databanks, we assumed this to be a wrong labelling. The feedback we got from the NCBI staff also supports our view. Therefore we used it to design primer and further comparison.

### Quality control and primer screening

Quality of the analyzed samples plays a major role in the correct interpretation of the results. Based on the morphological observations, in both *in vivo *derived and *in vitro *produced embryo samples, presumably good quality oocytes and preimplantation stage embryos were collected. In the subsequent procedures, in addition to our established quality RNA isolation procedures, the minus RT (reverse transcriptase) reaction during cDNA synthesis, and the design of most primers at the exon-exon junction enabled us to control the absence of contaminating genomic DNA. During the initial screening assays, similar cDNA dilutions from the pooled embryos were used and reaction conditions were optimised for each primer pair separately. Optimum primer concentrations were selected based on the absence of dimer and signal detection recognized by earlier C_T _values. The C_T _is defined as the number of cycles needed for the fluorescence to reach a specific threshold level of detection and is inversely correlated with the amount of template nucleic acid present in the reaction [35]. The majority of the selected candidate reference genes were detected in most preimplantation stages, but with various signal intensities. Using a similar low concentration template for all, the ten references (*Eef1e1, Polr2a, Ywhaz, Ppia, H2afz, Hprt1, Pgk1, G6pdx, Gapdh*, and *Actb*) and *POU5f1 *genes were detected at C_T _values below 33. The other three genes (*B2m, Ubc, and Tbp*) were detected at C_T _values above 33 with irregularities in amplification (at some of the stages where the transcripts of that particular gene were at lower concentration). As embryo materials are scarce at the preimplantation stages, we excluded the three genes detected with the latest C_T _values. Then, the ten candidate references and *POU5F1 *genes with the earlier signals (earlier C_T _values) were selected for further comparisons.

### Standards and PCR efficiency analyses

For all the selected candidate genes, melt curve analyses were performed at the end of PCR reactions. The specificity and integrity of the PCR products were confirmed by the presence of a single peak (See additional files 1, 2 and 3 as representatives). For the selected genes, the standard curves were deduced from four-fold serial dilutions of the five-pooled embryo cDNA preparations measured at five points. To ensure the comparability of PCR assays, three serial dilutions were made independently that enabled us to determine the C_T _values and PCR efficiencies of the individual assay and calculate the correlation between them. The assays for the selected candidate reference genes were characterized by a linear correlation coefficient (R^2^) that varied from 0.963 to 0.997 (average 0.986) and PCR efficiencies between 92.3% and 110.7% (average 97.5%), where as the assay for *POU5F1 *gene has 82.4% efficiency and 99.8% correlation (Table 3). Based on these results, the assays can be trusted and valid for the quantification of transcripts and further comparisons.

**Table 3:** Standard curve parameters for the candidate reference genes

Genes	Slope (m)	Intercept (b)	Efficiency (E)	Correlation (R^2^)
*Actb*	-3.37	21.90	98.0	97.9
*Gapdh*	-3.43	18.58	97.7	99.4
*Hprt1*	-3.25	22.56	103.1	98.9
*H2afz*	-3.52	18.85	92.3	99.4
*Ppia*	-3.51	16.07	92.6	99.7
*Eef1e1*	-3.48	20.50	94.0	97.2
*G6pdx*	-3.09	23.69	110.7	99.2
*Polr2a*	-3.38	22.10	97.7	96.3
*Pgk1*	-3.51	16.89	92.8	99.4
*Ywhaz*	-3.42	20.09	96.0	98.9
*POU5F1*	-3.83	17.32	82.4	99.8

### Reference genes profile analyses at different developmental stages

In order to select the best stable genes for normalization, expressions of the selected ten candidate reference genes were compared in different preimplantation stage embryos. For gene transcript quantification, five individual embryo cDNA preparations per stage were used in identical experimental procedures but optimised conditions for each gene amplification.

**Figure 1:**
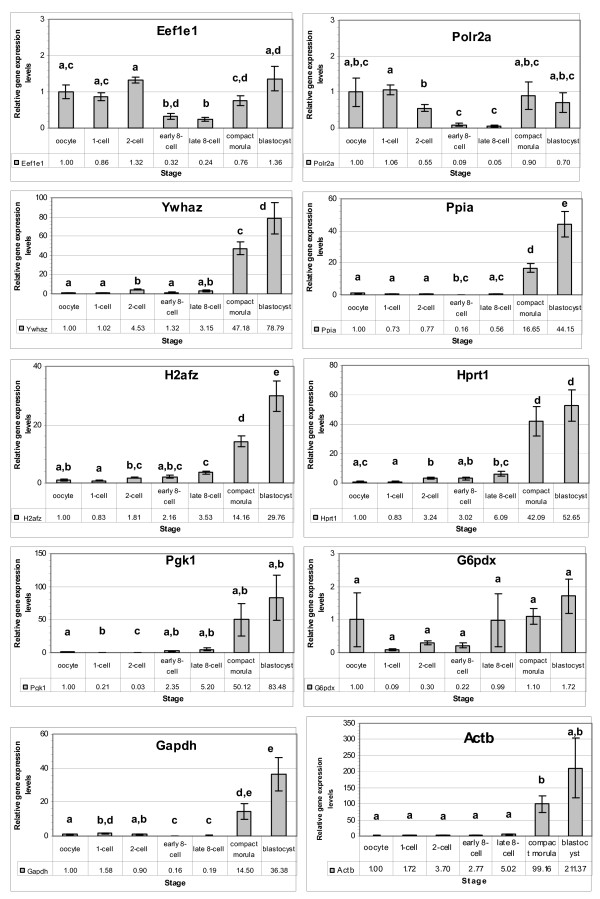
**Relative expression levels of selected candidate reference genes in the *in vivo *derived rabbit embryos. **The expression level at the oocyte stage was taken as a reference to calculate the relative levels of the other stages. Stages with different letters are significantly (P ≤ 0.05) different for the levels of the gene.

**Figure 2:**
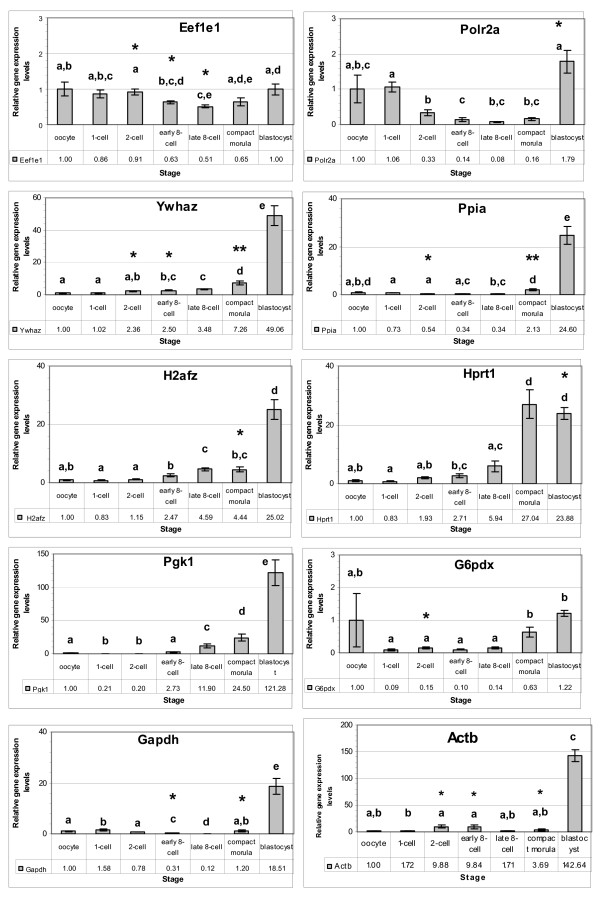
**Relative expression levels of selected candidate reference genes in the *in vitro *produced rabbit embryos.** The expression level at the oocyte stage was taken as a reference to calculate the relative levels of the other stages. Stages with different letters are significantly (P ≤ 0.05) different for the levels of the gene. An asterisk (*) indicates significant differences {(* = P ≤ 0.05) and (** = P ≤ 0.01)} with the *in vivo *levels of the same stage.

The ten candidate genes were detected in all developmental stages examined with various signal intensities. The oocyte stage was taken as a calibrator for all the genes and the relative expression levels at different developmental stages are shown in Figures 1 and 2.

Compared to the oocyte stage, the relative levels of most genes (*Eef1e1, Ppia, H2afz, Hprt, Pgk1 and G6pdx*) reduced at the 1-cell stage, while the relative levels of the remaining genes (*Polr2a, Ywhaz, Gapdh*, and *Actb*) increased in various intensities at this stage. The relative levels of some genes (*Eef1e1, Polr2a, Ppia, Gapdh, Actb (in vitro*) were transiently reduced to the minimum levels at the 8-cell stages, and increased thereafter. Despite differences in the embryo type (*in vivo *or *in vitro*), the above expression patterns remained similar (Figure 1 and Figure 2). In both *in vivo *derived and *in vitro *produced embryos, the relative levels of *H2afz *and *Hprt1 *genes showed a persistent increase in levels after fertilization. The gene *Ywhaz *(except 2-cell in vivo) also has the same profile.

### Comparative analyses in the *in vivo *derived and *in vitro *produced embryos

To examine the effects of embryo type (*in vivo *and *in vitro*) on gene expression stability, expression profiles of the selected candidate genes were compared in the *in vivo *derived and *in vitro *produced embryo samples.

Major pattern differences were not observed due to change in the embryo type (*in vivo *or *in vitro) *(Figures 1 and 2). However, the stage-by-stage comparisons revealed differential transcript levels between the two embryo types. At the 2-cell stage, *in vivo *produced embryos showed higher transcript levels for majority of the genes (except *Pgk1 *and *Actb*) compared to their *in vitro *counterparts, and differences were significant (p < 0.05) for some genes (*Eef1e1, Ywhaz, Ppia *and *G6pdx*). However, at the 8-cell stage, the *in vitro *samples showed higher transcript levels for majority of the genes, with significant difference (p < 0.05) for some (*Eef1e1, Ywhaz*, *Gapdh *and *Actb*). At the morula and blastocyst stages, the relative transcript levels of *in vivo *derived embryo samples were higher for most genes compared to the *in vitro *samples. These differences were significant (p < 0.01 for *Ywhaz *and *Ppia*, and p < 0.05 for *H2afz*, *Gapdh *and *Actb*) at the morula stage and (p < 0.05 for *Polr2a *and *Hprt1*) at the blastocyst stage (Figure 2).

### Gene expression stability analyses

**Figure 3:**
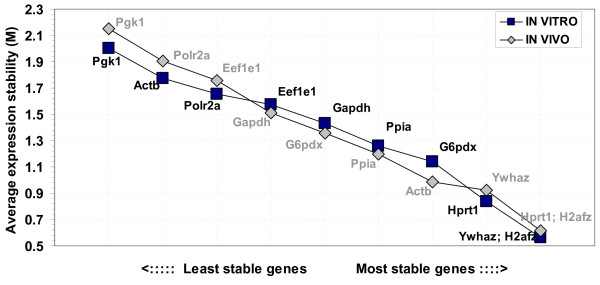
Average gene expression stability values of the candidate reference genes in the *in vivo *derived and *in vitro *produced embryo samples as calculated by geNorm software and ranking made based on the relative stability values.

The profiles of candidate genes were analysed and the expression stability measure values (M) were calculated using the geNorm software [36]. Following the procedures of the software, the least stable genes were determined (by higher M values) and continuously excluded to recalculate the M values for the rest. Finally all genes were ranked based on the calculated M values and the three most stable genes (with the lowest M values) were selected. Accordingly, for the *in vivo *derived samples, the genes *H2afz *and *Hprt1 *were found to be the most stable, followed by the genes *Ywhaz, Actb, Ppia, G6pdx, Gapdh, Eef1e1*, *Polr2a *and *Pgk1 *in their order of appearance. Similar analyses for the *in vitro *samples selected the genes *H2afz *and *Ywhaz *as the most stable genes, followed by *Hprt1, G6pdx, Ppia, Gapdh, Eef1e1, Polr2a, Actb *and *Pgk1 *in their order of appearance. Generally, differences in the embryo type (*in vivo *or *in vitro*) had a minor rearrangement effect on the stability order for majority of the genes examined. The gene *Actb *showed wider stability range between the embryo types with better stability in the *in vivo *samples compared to the *in vitro *(Figure 3).

### Expression profiles of *POU5F1 *gene in rabbit preimplantation stage embryos

**Figure 4:**
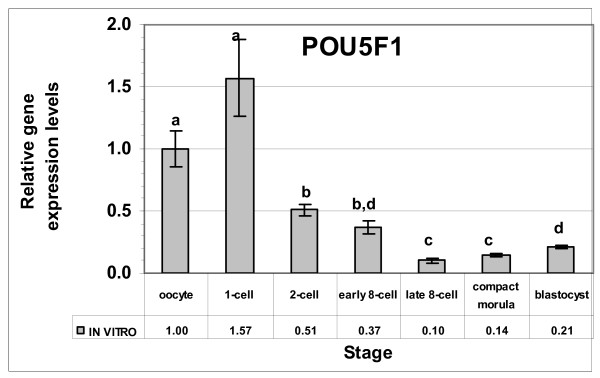
**Relative expression levels of the *POU5F1 *gene in the *in vitro *produced preimplantation stage rabbit embryos.** The expression at the oocyte stage was taken as a reference to calculate the relative levels of the other stages. Stages with different letters are significantly (P ≤ 0.05) different for the levels of the gene.

To evaluate the performances of the newly selected reference genes, the normalization factor (calculated from the geometric averages of the three selected genes) was calculated. The expression of *POU5F1 *gene was quantified in the *in vitro *produced rabbit oocytes and embryo samples and the results were normalized by the newly calculated factor. The expression at the oocyte stage was used as a calibrator to calculate the relative expression levels in the different developmental stages (Figure 4). Based on analyses, the *POU5F1 *gene was expressed in all developmental stages with various signal intensities. Comparatively higher expression levels were observed at the oocyte and zygote stages, and declined gradually to the lowest levels at the 8-cell stage. However, starting from the morula stage, the levels increased continuously to the blastocyst stage.

## Discussion

The number of studies revealing the importance of normalization has increased recently and, as a result, the search for study-specific appropriate reference genes has gained momentum. This can be seen in the rising number of relevant publications. In this study, we selected a large number of reference genes and compared their expression in the rabbit oocytes and different preimplantation stage embryos that were derived *in vivo *or produced *in vitro*. After detailed analyses, here we show the selection of the three most stable reference genes in rabbit oocytes and embryos that can be used for normalization.

Rabbits have many advantages [24,27], including a convenient reproductive pattern (non-seasonal breeding, induced ovulation and short gestation period of 31 days), the possibility of keeping them in conventional housing in an indoor facility, the lower cost of procurement and handling compared to large animals. Moreover, they have sizeable milk production that allows their use as test animals for therapeutic protein expression in milk. A combination of all these factors has led to the use of rabbits as experimental models and their use in gene expression studies. Moreover, the studies of genetic reprogramming in rabbit embryos [37-41,27], in which our laboratory is also taking part, are surging.

As gene expression results depend on the type of normalizer gene used [42,17], finding appropriate reference genes is timely, and will help toward the correct interpretation of the experimental results. Finding appropriate reference genes implies careful selection of stable genes evaluated for expression stability. Differences arising from the quality and quantity of input RNA, efficiencies of reverse transcription reaction and handling errors can be accounted by normalizing the expression of a target gene to the reference. This allows the direct comparison of normalized expression values between samples [43].

Earlier suggestions to use RNA mass quantity and adding exogenous templates for normalization has been challenged for the inherent technical problems hindering its wider adoption [36,9,19,12]. Moreover, adding exogenous template will compete with endogenous sequences for primers and nucleotides during the PCR reaction [9]. The use of endogenous reference genes for normalization is the widely used approach in most applications. It has gained acceptance over time due to its biological relevance, and its consideration of various errors during the process. Moreover, compared to adding exogenous templates to the samples, it has no extra procedures for its application [19]. To address this issue, the use of individual embryos compared to the pooled ones was in line with earlier findings [44-46] that indicated the significance of using individual samples. It can enable accurate statistical analyses, identify more biological variations and subtle gene expression changes.

Most of the genes (*Eef1e1, Polr2a, Ppia, Gapdh *and *Actb in vitro) *had the respective lowest levels at the 8-cell stage. This transcript depression at the 8-cell stage coincides with the lag in development (developmental block) occurring during the maternal to zygote transition (MZT) in rabbit embryos [47-49]. The morphological and molecular variations between embryos of different mammalian species have been discussed [3]. The effects of culture and treatment conditions on the rabbit preimplantation stage embryos have also been described earlier [50-52]. In this study, the comparative transcript levels of the same gene in different embryo types (*in vivo *vs. *in vitro*) were described. The profile shows transcript level variations and substantiates the fact that *in vitro *conditions, in general, are sub-optimal and influence gene expression levels. In our earlier mouse study [12], we have indicated the effects of embryo type on the stability and selection of reference genes. As far as we know, such comparisons and selections were not made for rabbit gene expression studies. Therefore, we made comparison of large number of commonly used reference genes and selected the most stable ones for normalization. Thus, this study is timely and the recommendations derived from the study can be widely applicable for rabbit embryo studies elsewhere, as most rabbit embryo studies deal with both or either of the *in vivo/in vitro *models.

Despite the traditional ways of using a single reference gene for normalization, the approach has been frequently criticized in a number of studies [28,30,43,17]. The implications of using an inappropriate reference gene have already been discussed [17]. Another elegant study [36] also demonstrated the error related to using a single reference gene and proposed to calculate a normalization factor based on the geometric averages of at least three carefully selected stable reference genes. In this study, detailed comparisons of the expression levels in different developmental stages and embryo types (*in vivo *vs. *in vitro*) indicated the comparative stable expression of the genes *H2afz, Ywhaz *and *Hprt1*. This was also further confirmed by the geNorm expression stability analyses (Figure 3). The selected constitutive genes were stable in both *in vivo *and *in vitro *conditions with slight variations in the order of stability (Figure 3). This variation might be attributed to the differences in expression levels with change in embryo type. Our earlier mouse study [12] has also indicated a shift in the stability order with the changes in embryo type.

In the current study, variations of gene stability values between the *in vivo *and *in vitro *comparisons were narrower than in the earlier mouse study. This may indicate better *in vitro *culture conditions of rabbit embryos than the *in vitro *mouse embryo culture. Moreover, the comparative stability of *H2afz *and *Hprt *genes were also shown in our previous mouse study. Although the gene *Ppia *was a preferred reference gene in the earlier studies [12,53], it is not among the best in this study. This might be due to species differences or the presence of other better performing genes. In earlier studies, some genes (including *Actb *and *Gapdh*) were assumed as universal reference genes, without further evaluation, and used individually for normalization of gene expression data [19]. The current result in rabbit embryos and our earlier mouse study [12] clearly indicated the inappropriateness of some of the widely used reference genes for normalization, at least under the conditions examined, and reinforce the recommendation to evaluate study-specific reference genes before using them for normalization.

To evaluate the performances of the newly selected reference genes, the expression values of *POU5F1 *gene was normalized by the calculated new normalization factor. After normalization, the observed pattern closely resembled the profiles of *POU5F1 *orthologue genes. Since *POU5F1 *orthologue genes, including human, bovine and porcine, are highly conserved, a similar role has been suggested for *POU5F1 *in all mammals [54]. In line with our finding of the *POU5F1 *profile, earlier studies in human [55] and bovine [56] embryos detected the expression of *POU5F1 *throughout all preimplantation stages. The decline of *POU5F1 *levels during the MZT stage and continuous increase after compaction was also observed in the earlier mouse [57] and human [54] embryo studies. Therefore, the normalized *POU5F1 *gene profile revealed the conserved pattern and further confirmed the suitability of the selected reference genes.

## Conclusion

Our study, for the first time, revealed a detailed reference gene validation and selection for rabbit preimplantation stage embryo studies. The outcomes indicated the possibility of using the same selected genes for both *in vivo *derived and *in vitro *produced embryo gene expression studies. Although transcript level variations were observed between individual embryos analyzed for the same gene, the expression patterns were almost similar. Based on detailed analyses of the results, including the pattern and ranking with geNorm, the genes *H2afz, Ywhaz *and *Hprt1 *were found to be the most stable. We also believe that the number of reference genes used for normalization depends on several factors [36]. However, using the geometric averages of the above three genes is preferred for accurate gene expression results in rabbit oocytes and preimplantation stage embryo studies. The appropriateness of these genes irrespective of the embryo type, and the conserved patterns of the *POU5F1 *gene after normalization, further confirm the suitability of these genes.

## Methods

All chemicals, unless stated otherwise, were purchased from Sigma-Aldrich Chemical Inc. (St. Louis, USA).

The animal experiments were executed in full compliance with European and Hungarian laws and regulations, and were approved by the Agricultural Biotechnology Center, Gödöllö, Animal Experimentation Committee.

### Oocyte collection

Hycole hybrid female rabbits were induced to superovulate by administration of 120 IU pregnant mare serum gonadotropin (PMSG, Folligon^® ^Intervet, The Netherlands) *i.m*. and 72 hours later, these animals were injected *i.v*. with 170 IU human chorionic gonadotropin (hCG, Choragon^® ^Richter Gedeon Rt., Hungary). Donor females (does) were slaughtered at 16 hours post hCG administration and the oocytes were flushed from the ampullae of the oviducts with M2 medium. The cumulus layer was then removed using 0.1% hyaluronidase in M2 medium, and the denuded metaphase 2 stage oocytes (confirmed by the presence of a single polar body) were collected individually for RNA isolation.

### Zygote collection

Hycole hybrid female rabbits were induced to superovulate by administration of PMSG, and 72 hrs later, and shortly before mating, with hCG hormones as described earlier. Each injected female was mated with a male of proven fertility (buck) from the same breed. Donor females were slaughtered at 20 hours post hCG administration and the zygotes were flushed from the oviduct with M2 medium. The flushed zygotes were evaluated morphologically under the microscope and those with two pronuclei, two polar bodies and compact cytoplasm were selected for the experiment.

### *In vitro *embryo production

The *in vitro *embryos were produced by further culturing the zygotes until the required developmental stages. For this, the zygotes were cultured in 50-μl EBSS (Earle's Balanced Salt Solution) complete drops [56] under mineral oil and incubated at 38.5°C in humidified atmosphere containing 5% CO_2 _in air. The embryos were cultured until the required developmental stages, and five embryos each at the 2-cell (26 hrs), 8-cell early (44 hrs), 8-cell late (54 hrs), morula (68 hrs), and blastocyst (103 hrs) stages were collected individually.

### *In vivo *embryo production

The procedures of superovulation and mating were as described earlier. For collection of *in vivo *derived embryos, donor females were slaughtered at respective times (hours post hCG administration) and the embryos at the 2-cell (26 hrs), 8-cell early (41 hrs), 8-cell late (47 hrs), morula (62 hrs), and blastocyst (98 hrs) stages were collected individually.

During sample collection, the denuded oocytes and different developmental stage embryos were washed three times with and collected individually in 2-μl RNase-free water for storage at -80°C until RNA extraction.

### RNA isolation and cDNA synthesis

The procedures of RNA isolation and cDNA synthesis were as described in detail earlier [15]. Briefly, messenger RNA was extracted individually from five embryos per developmental stage and embryo type (*in vivo *or *in vitro*), using Dynabeads^® ^mRNA DIRECT™ Micro Kit (Dynal A.S, Oslo, Norway), following the manufacturer's instructions. The individually frozen embryos were lysed and incubated with pre-washed magnetic Dynabeads that can base pair with poly (A) tails of mRNA molecules. After hybridisation and subsequent repeated washes with buffers, the RNA was eluted in RNase-free water and reverse transcribed into cDNA, using M-MLV RT kit (Invitrogen, Carlsbad, USA) in a final 20-μl reaction volume. Minus RT reactions were performed to check the absence of contaminating residual DNA. The cDNA synthesis reactions were carried at 42°C for 1 hour, followed by heat inactivation of the enzyme at 75°C for 10 minutes.

### Primer design and sequence analyses

A total of 14 genes, most commonly used and considered as reference for normalization by the earlier studies, were selected for evaluation throughout the different embryo developmental stages and embryo types (*in vivo *and *in vitro*). Moreover, *POUF1 *gene was also used for evaluating the selected reference genes. Primers were designed and optimised prior to initial screening and quantitation experiments. Comparison and screening of primers were carried out using optimised protocol for each primer. Template cDNA from the same source was used, and finally those with earlier signals were selected for further quantification. For these genes (Table 1), the expected sizes of the products were confirmed by gel electrophoresis on a 2% agarose gel. The PCR products were also cloned (TOPO TA cloning kit, Invitrogen), and sequenced for confirmation.

### Real time PCR reaction conditions and analyses

The details of real time PCR conditions for each gene were as described in Table 2. During quantification of the transcripts, the assay for each gene consisted of five replicates per stage, both *in vivo *derived and *in vitro *produced oocytes and preimplantation stage embryo samples, negative and positive controls. All genes were compared from the same stock to avoid inter-assay template variations, and all quantifications were performed consecutively without interruption. Each sample in a run consisted of 0.08 embryo equivalent cDNA template, 200–500 nM of each primer (Table 2), and 50% SYBR^® ^Green JumpStart™ Taq ReadyMix™ in 15-μl reaction volume. The reaction conditions were template denaturation and polymerase activation at 95°C for 2 min followed by 45 cycles of 95°C denaturation for 15 sec, 57°C to 68°C annealing and extension for 45 sec. All reactions were carried out using the Rotor-Gene™ 3000 real time PCR machine (Corbett Research, Mortlake, Australia), and the results were analysed with the integrated Rotor-Gene software (version 6.1). At the end of PCR reactions, melt curve analyses were performed for all the genes. For calculating PCR efficiencies, standard curves were generated from assays made with four fold serial dilutions of 5-pooled blastocyst cDNA preparations. To ensure the compatibility of PCR assays, three independent serial dilutions were made that enabled us to determine the C_T _values and PCR efficiencies of the individual assay and calculate the correlation between them. PCR efficiencies (E) were calculated with the equation

E = (10 [-1/slope]-1) × 100.

### geNorm and expression stability analyses

Analyses of the gene expression stability over the different embryonic stages and types (*in vivo *vs. *in vitro*) were performed using the geNorm software [36]. The analyses relies on the principle that the expression ratio of two ideal internal control genes is identical in all samples, regardless of the experimental condition or cell type, and determined as the standard deviation of the logarithmically transformed expression ratios [36]. The internal control gene stability measure value (M) was calculated as the average pair-wise variation of a particular gene with respect to the rest of the genes, and ranking was made based on these values. The lower the M value, the more stable the expression of the gene under consideration. The most stable reference genes were identified by stepwise exclusions of the least stable gene and recalculating the M values with the rest genes.

## Authors' contributions

SM conceived the experiment, performed the experimental design, all the molecular biology analyses, including experiment execution, data analyses and interpretation, and was the primary author of the manuscript. ABG participated in the experimental design, performed primer design, data analyses, statistical tests and participated in manuscript preparation. ZP prepared *in vivo *and *in vitro *embryos for the experiment. AD supervised the study design, execution, analysis, and approved the final version. All authors read and approved the manuscript.

## Supplementary Material

Additional file 1 Supplementary figure 1. Representative melt curve and cycle threshold analyses of the gene *Ywhaz*. Click here for file

Additional file 2 Supplementary figure 2. Representative melt curve and cycle threshold analyses of the gene *H2afz*. Click here for file

Additional file 3 Supplementary figure 3. Representative melt curve and cycle threshold analyses of the gene *Hprt*. Click here for file
