# Supplementary figures and images for: Expression profiles of the pluripotency marker gene POU5F1 and validation of reference genes in rabbit oocytes and preimplantation stage embryos
Source: BMC Mol Biol. 2008 Jul 28;9:67. doi: 10.1186/1471-2199-9-67 (PMC2507718; doi:10.1186/1471-2199-9-67)

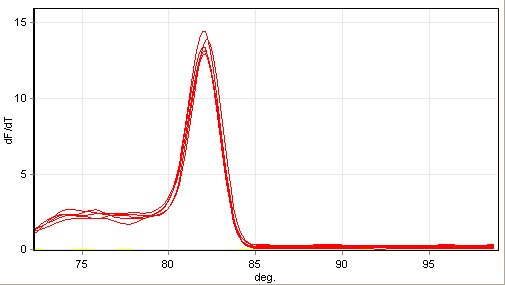


Additional file 1. Representative melt curve and cycle threshold analyses of the gene *Ywhaz*.

Supplement: Additional file 1 — Supplementary figure 1. Representative melt curve and cycle threshold analyses of the gene Ywhaz. [file 1471-2199-9-67-S1.doc]
